# Paraganglioma-induced reverse takotsubo syndrome treated with extracorporeal membrane oxygenation in a young patient with a history of malignancy: a case report

**DOI:** 10.1093/ehjcr/ytad591

**Published:** 2023-11-24

**Authors:** Steven C Ajluni, Rafey Feroze, Sylvia L Asa, Varun Sundaram

**Affiliations:** Department of Medicine, University Hospitals Cleveland Medical Center, Cleveland, OH, USA; Harrington Heart and Vascular Institute, University Hospitals Cleveland Medical Center, Cleveland, OH, USA; Department of Pathology, Case Western Reserve University School of Medicine, Cleveland, OH, USA; Harrington Heart and Vascular Institute, University Hospitals Cleveland Medical Center, Cleveland, OH, USA

**Keywords:** Case report, Reverse takotsubo cardiomyopathy, Paraganglioma, Cardiogenic shock, VA-ECMO, Extracorporeal membrane oxygenation

## Abstract

**Background:**

Reverse takotsubo-like cardiomyopathy (rTCC) is a rare type of stress-induced cardiomyopathy associated with catecholamine surges. Reverse takotsubo-like cardiomyopathy is characterized by basal and mid-ventricular hypokinesis with apical sparing. Paragangliomas are catecholamine-secreting neuroendocrine tumours outside the adrenal gland that can cause palpitations, hypertension, and rarely cardiomyopathy. In cases of occult paraganglioma, catecholamine-induced rTCC can be rapidly reversed with adequate haemodynamic support.

**Case summary:**

A 28-year-old woman with a history of cervical cancer, ovarian insufficiency, and preeclampsia presented to the emergency department with nausea, vomiting, and chest pain. The patient was initially tachycardic, tachypnoeic, and hypotensive. On exam, she was in distress with diffuse rales and cool extremities. Electrocardiogram showed sinus tachycardia to 147 b.p.m. and lateral ST depression in V4 and V5. Troponin was elevated to 13 563 ng/L. An echocardiogram showed severely reduced left ventricular ejection fraction (LVEF) with hypokinesis of the basal segments and apical sparing, identified as rTCC. Computed tomography of the abdomen showed a 3.6 × 2.7 cm right adrenal mass. The patient rapidly developed respiratory failure and was subsequently intubated, sedated, and initiated on vasopressors. In the setting of cardiogenic shock refractory to vasopressor support, the decision was made to cannulate for venoarterial extracorporeal membrane oxygenation (VA-ECMO). Plasma and urine metanephrines were elevated. After 5 days, the patient’s LVEF recovered to her baseline, and the rTCC had resolved. The patient’s hypertension was managed with gradual alpha-blockade, and she subsequently underwent successful adrenalectomy on Day 44.

**Discussion:**

An occult paraganglioma should be considered when rTCC pattern is identified. The pathophysiology of paraganglioma-mediated catecholamine surges predisposing to rTCC is unclear. Potential mechanisms for rTCC include oestrogen deficiency, catecholamine cardiotoxicity, and coronary artery spasm. The VA-ECMO is an increasingly used modality to provide haemodynamic support to patients with refractory cardiogenic shock.

**Learning points**

- To develop a differential diagnosis when cardiogenic shock with a reverse takotsubo cardiomyopathy (rTCC) pattern is identified.
- To understand the management strategy when presented with a reversible cardiomyopathy secondary to paraganglioma-induced catecholamine surge.
- To describe the clinical significance of extra-adrenal paragangliomas and the connection between oestrogen deficiency and rTCC.

## Introduction

Reverse takotsubo-like cardiomyopathy (rTCC) is a stress-induced cardiomyopathy characterized by basal and mid-ventricular hypokinesis with apical sparing.^1^ Reverse takotsubo-like cardiomyopathy has been reported with excess catecholamine release and can present with decreased left ventricular (LV) function with preserved cardiac output (CO) to fulminant cardiogenic shock. We present a case of cardiogenic shock with a rTCC pattern in the setting of a peri-adrenal paraganglioma (PGL)-induced catecholamine surge successfully managed with venoarterial extracorporeal membrane oxygenation (VA-ECMO).

Paragangliomas are non-epithelial neuroendocrine neoplasms arising from the sympathetic and parasympathetic nervous systems.^2^ Sympathetic paraganglia are most often located in the abdomen; the largest are the bilateral adrenal medullas and the organ of Zuckerkandl.^2^ Of sympathetic PGLs, 80% are found in the adrenal medulla and are known as pheochromocytomas. PGLs are frequently associated with a causative genetic syndrome.^3^

Paragangliomas have been associated with stress-induced cardiomyopathy.^4^ Among the takotsubo subtypes, PGLs more commonly cause a classical takotsubo cardiomyopathy (TTC) with apical hypokinesis compared with an inverted takotsubo with basal hypokinesis and apical sparing known as rTCC.^1^ Among all patients who presented with elevated troponins and suspected acute coronary syndrome, TTC is found in about 2% of patients. Of these patients with TTC, rTCC has been described anywhere from 1 to 23% of TTC cases.^5^ In a review of 38 cases of pheochromocytoma-associated cardiomyopathy, about one-third of cases presented with rTCC and were associated with higher complication rates than TTC.^6^

## Summary figure

**Figure:**
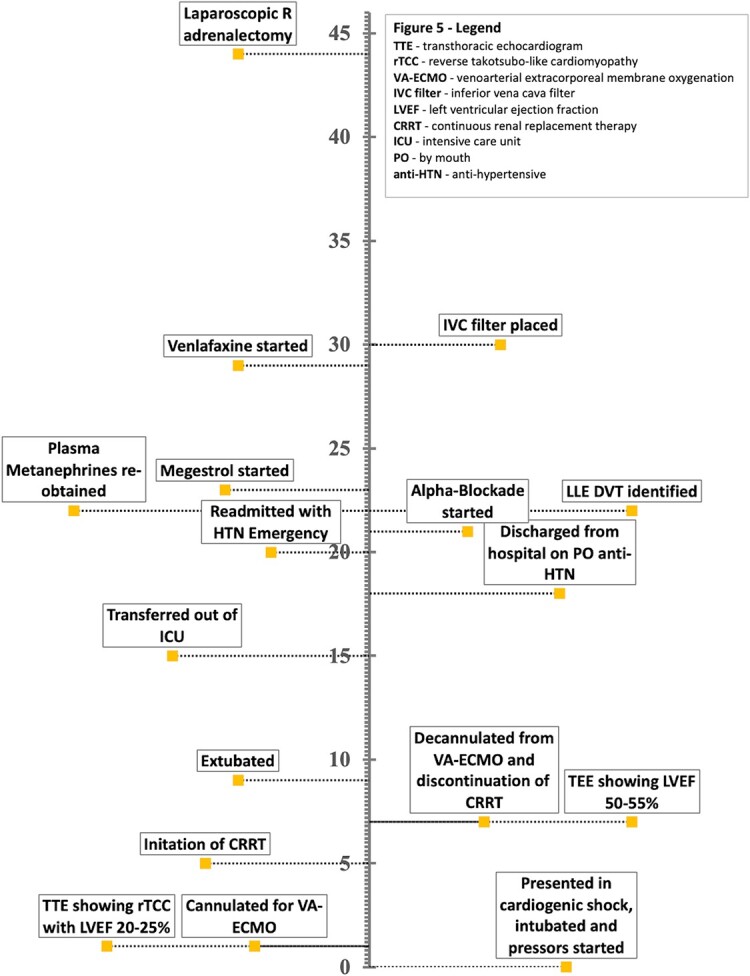


## Case presentation

**Figure 1:**
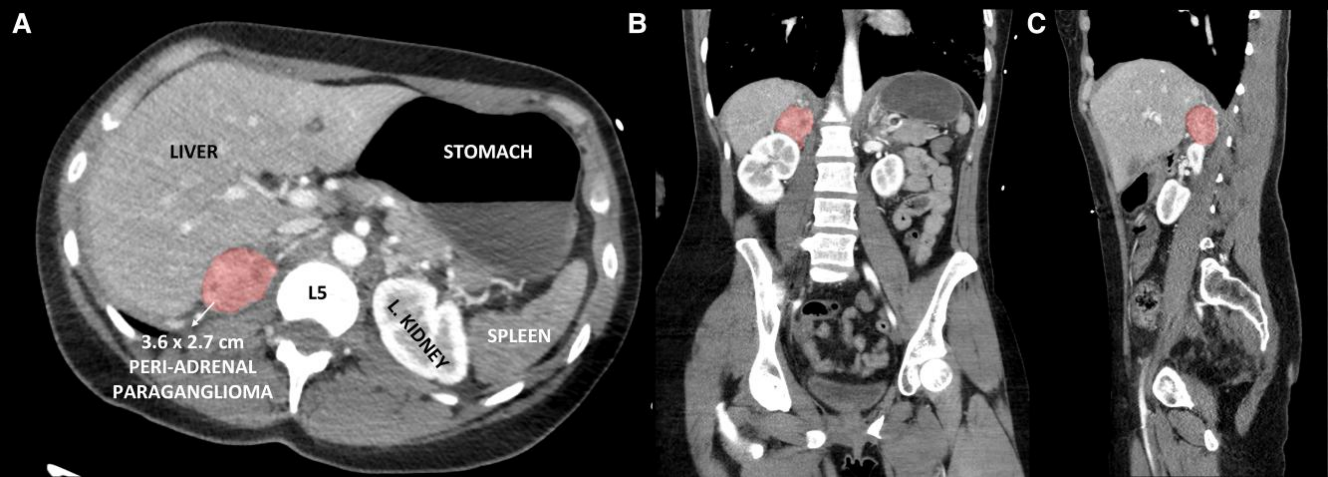
Computed tomography image of the 3.6 × 2.7 cm right-sided adrenal mass in coronal (*A*), axial (*B*), and sagittal (*C*) cuts.

A 28-year-old female with a history of preeclampsia and treated cervical cancer resulting in ovarian insufficiency presented to the emergency department with chest pain, nausea, vomiting, and diaphoresis. She was tachycardic, tachypnoeic, and hypotensive with diffuse bilateral rales and cold extremities. Cardiovascular examination was significant for tachycardia with a normal S1 and S2; no murmurs were appreciated. Initial lab values are outlined in *Table 1* and were significant for leucocytosis, acidemia, hepatocellular transaminitis, and troponinaemia. Electrocardiogram revealed lateral ST depression in V4 and V5 (see Supplementary material online, *Figure S1*). Computed tomography (CT) imaging did not reveal a pulmonary embolism and showed bilateral lower lobe opacities along with a 3.6 × 2.7 cm right adrenal mass (*Figure 1*). Coronary pathology, such as myocardial infarction, dissection, or thromboembolism, was considered. However, coronary angiography was deferred given the demographics (young age, absence of risk factors, and no family history) and clinical presentation (rapid respiratory failure, adrenal mass). Sepsis was considered given pulmonary infiltrates and rapid respiratory failure but ultimately ruled out given limited supporting evidence. She was intubated, started on vasopressor support, and life flighted to an ECMO-equipped facility.

**Table 1:** Initial laboratory data from the day the patient was admitted

Lab test	Lab values
White blood cell count, mm^3^	12 600 (4400–11 300)
Haemoglobin, g/dL	10.7 (12–16)
Platelet count, per microlitre	132 000 (150–450)
International normalized ratio, plasma	2.4 (0.9–1.1)
Prothrombin time, plasma, s	28.0 (9.8–13.4)
Activated partial thromboplastin time, s	133 (26–39)
Sodium, mmol/L	143 (136–145)
Potassium, mmol/L	5.4 (3.5–5.3)
Bicarbonate, mmol/L	20 (21–32)
Blood urea nitrogen, serum, mg/dL	36 (6–23)
Creatinine, serum, mg/dL	1.84 (0.50–1.05)
Alanine aminotransferase, serum, U/L	>5000 (7–45)
Aspartate transaminase, serum, U/L	8147 (9–39)
Alkaline phosphatase, serum, U/L	31 (33–110)
Bilirubin, serum total, mg/dL	0.6 (0.0–1.2)
d-Dimer, ng/mL	795 (<500)
Lactic acid, mmol/L	8.0 (0.4–2.0)
Pro-BNP, pg/mL	211 (0–99)
Troponin I, high sensitivity, ng/L	13 563 (0–34)
pH	7.12 (7.35–7.45)

**Figure 2:**
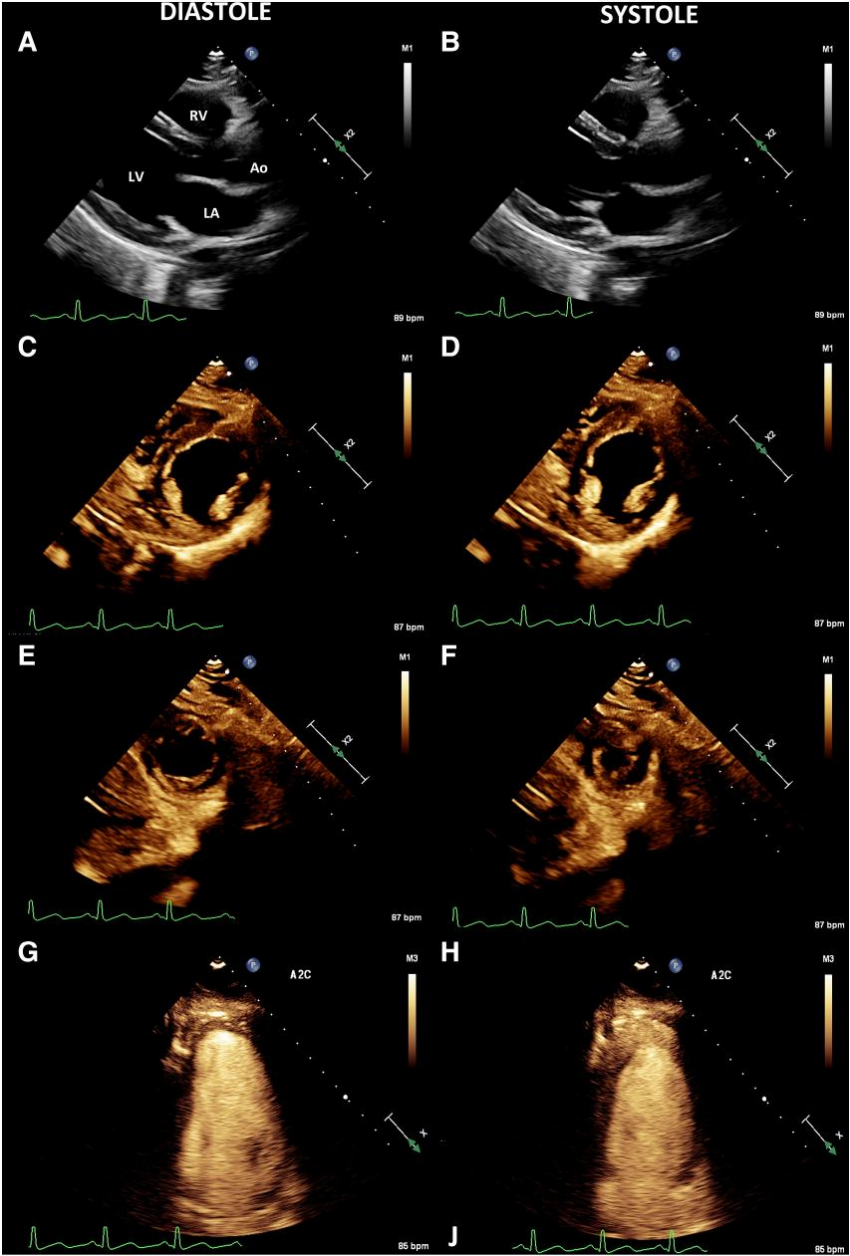
Transthoracic echocardiogram images (*A–H*) showing a reverse takotsubo-like cardiomyopathy pattern with preserved function of the left ventricular apex (*F*) and hypokinesis of the basal and septal segments (*B* and *D*).

Transthoracic echocardiogram (TTE) on Day 1 showed LV ejection fraction (LVEF) of 20–25% with a hypokinetic base and preserved apex, suggestive of rTCC (*Figure 2*). Due to end-organ hypoperfusion refractory to pressors, the decision was made to cannulate for VA-ECMO on Day 1. Extracorporeal membrane oxygenation weaning trials were attempted daily, and upon LVEF recovery, ECMO was decannulated on Day 7. Due to complete LVEF recovery, a coronary aetiology was considered less likely. Continuous renal replacement therapy was required for volume overload and azotaemia from Days 5 to 7. She was extubated on Day 9 and transitioned out of the intensive care unit (ICU) on Day 15. The secretory capacity of the adrenal mass was investigated with plasma and urine metanephrines and normetanephrines, which were elevated, but difficult to interpret in the setting of high-dose exogenous catecholamine administered in the peri-shock period (*Table 2*). On the regular nursing floor, the patient demonstrated stability with controlled blood pressure and improved renal function. On Day 18, the patient was discharged with close outpatient follow-up with endocrine for further management of her adrenal mass.

**Table 2:** Endocrinologic laboratory data

Lab test	Lab values
Metanephrine, plasma, nmol/L	1.5 (<0.5)
Normetanephrine, plasma, nmol/L	15 (<0.9)
Metanephrine, urine, µg/L	405
Metanephrine, urine 24 h, µg/24 h	565 (36–209)
Normetanephrine, urine, µg/L	1848
Normetanephrine, urine 24 h, µg/24 h	2580 (95–449)
17-Hydroxyprogesterone, serum, ng/dL	25 (38–583)
17-Hydroxypregnenolone, serum, ng/dL	39 (post-menopausal <45)

The patient was readmitted on Day 20 with dyspnoea, chest tightness, and bibasilar crackles. She was in hypertensive emergency and flash pulmonary oedema with preserved LV function. Repeat imaging showed an unchanged 3.6 × 2.7 cm right adrenal mass. Given that she had not been on exogenous catecholamines recently, urine metanephrines and normetanephrines were obtained again and were elevated. A deep vein thrombosis (DVT) was identified in the left lower extremity on Day 22, the same side as the recent VA-ECMO cannulation. Oxygenation improved following blood pressure control with prazosin. After sufficient alpha-blockade with prazosin, blood pressure remained elevated, necessitating the addition of labetalol, chosen for its alpha- and beta-blocking properties. Despite an escalation of alpha-blockade, the patient had early morning blood pressure spikes associated with hot flashes. These symptoms of chronic ovarian insufficiency potentially contributed to PGL-mediated catecholamine surges predisposing to rTCC. She was started on megestrol and venlafaxine to manage the vasomotor symptoms of ovarian insufficiency. Given planned adrenal mass resection, she received a retrievable inferior vena cava (IVC) filter on Day 30 so that anticoagulation could be safely stopped prior to surgery. On Day 44, she underwent laparoscopic right adrenalectomy; pathology confirmed the diagnosis of peri-adrenal PGL. At follow-up, genetic testing was negative, and the patient remained asymptomatic. Upon further history obtained from the family, the patient had a 2-year history of flushing, tachycardia, palpitations, and dyspnoea not associated with exertion prior to this presentation. At a 1-year follow-up, the patient’s echocardiogram showed a preserved LVEF without evidence of wall motion abnormalities or valvular disease.

## Discussion

Our patient developed rTCC, resulting in fulminant cardiogenic shock secondary to peri-adrenal PGL-induced catecholamine surge, requiring VA-ECMO support.

Paragangliomas present rarely with severe cardiomyopathy causing cardiogenic shock. In cases of cardiogenic shock refractory to inotropes and vasopressors, VA-ECMO has been used to provide haemodynamic support and concomitant gas exchange.^7^ Catecholamine-induced TTC and rTCC have a well-documented reversible pattern, thus making VA-ECMO a particularly useful modality as a bridge to myocardial recovery.^1,5,7^

Cardiomyopathy associated with catecholamine surges has an unclear mechanism.^1^ The apex has the highest concentration of beta-adrenergic receptors, making it uniquely susceptible to high levels of catecholamines.^1^ Catecholamines are believed to cause cardiotoxicity via myocardial necrosis, neutrophil infiltration, and fibrosis.^5^ However, this does not explain the inverted pattern seen in rTCC. Alternative mechanisms include coronary artery spasm and coronary microvasculature impairment.^5^ Emotional and physical triggers, such as depression, subarachnoid haemorrhage, and oestrogen deficiency, have been associated with TTC and its various subtypes (including rTCC).^8^

Specifically, rTCC has been associated with oestrogen deficiency.^5^ Oestrogen and its receptor, estrogen receptor (ER), play a role in cardioprotection.^9^ In animal models of cardiac ischaemia, activation of the ER receptor was found to reduce cardiomyocyte apoptosis, inflammation, and oxidative stress, as well as induce vasodilation and increase neovascularization.^10^ Our patient had iatrogenic oestrogen deficiency from prior cervical cancer treatment. While it may have contributed to her malignant presentation, the precise role that oestrogen deficiency plays in the development of rTCC and cardiogenic shock is unclear.

**Figure 3:**
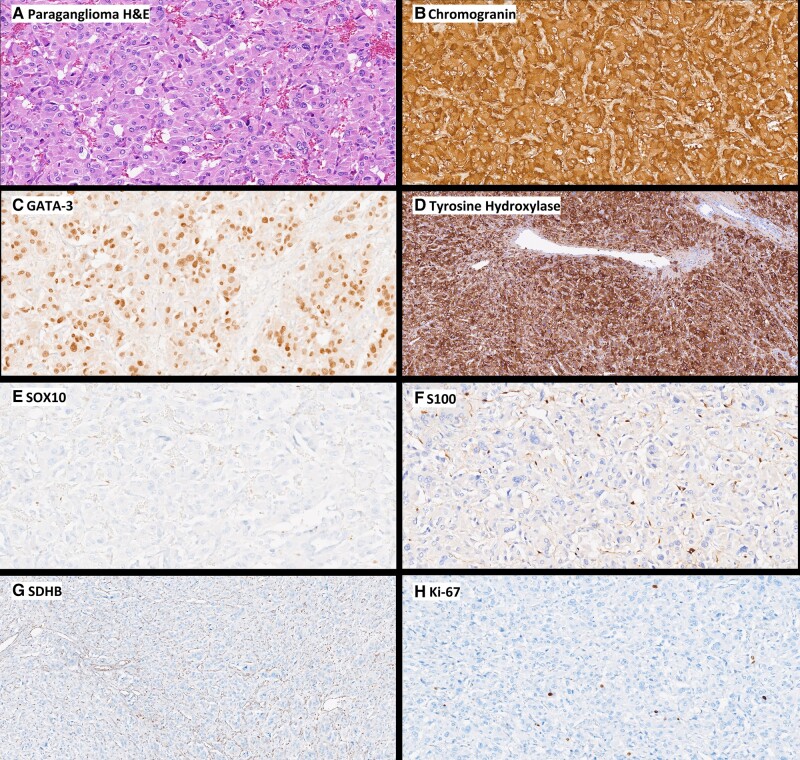
Pathologic specimens of the right-sided peri-adrenal mass. (*A*) Haematoxylin and eosin showing the nesting architecture of the tumour. (*B*) Tumour cells express chromogranin. (*C*) Tumour cell nuclei stain for GATA-3. (*D*) Tumour cells have cytoplasmic positivity for tyrosine hydroxylase. (*E*) SOX10 stains the nuclei of sustentacular cells. (*F*) S100 highlights sustentacular cells. (*G*) SDHB loss in tumour cells; the stromal cells provide an internal positive control. (*H*) The Ki-67 labelling index was <1%.

The patient’s pathology (*Figure 3*) demonstrated strong expression of chromogranin, GATA-3, and tyrosine hydroxylase, which are markers for PGLs.^3^ Ki-67, SOX10, and S100 are markers used to assess the risk of tumour recurrence/metastasis and indicate a low likelihood of a biologically aggressive PGL.^3^ While the peri-adrenal nature of this tumour increased our suspicion for an endocrinopathy as the cause of rTCC, PGLs can also arise elsewhere in the body, requiring clinical vigilance when a tumour is identified in the context of stress cardiomyopathy.^2^ Additionally, the genetic association of PGLs cannot be ignored, as 40% of these tumours are hereditary.^2^ Our patient’s tumour showed loss of SDHB, suggesting SDHx-related disease; however, her subsequent genetic testing was negative for SDHx mutations. A potential explanation for the incongruous genetic and pathologic data is a post-zygotic epimutation of one of the SDH genes.^11^

In an effort to characterize commonalities between the present case and others in the literature, we identified seven published cases of extra-adrenal PGL or pheochromocytoma causing rTCC managed with VA-ECMO^12–18^ (see Supplementary material online, *Table S1*). All patients except for one were female, and all but one were over the age of 45. The degree of ovarian dysfunction in these patients was not reported, but it is plausible that a component of oestrogen deficiency was present in these patients. Five of seven patients experienced cardiac arrest during their course, including the present patient, and all patients showed complete LVEF recovery following VA-ECMO. Five of seven patients had elevated urine or serum catecholamines (metanephrine or normetanephrine), which are confounded by concomitant vasopressor administration in the peri-shock period.

## Conclusion

In summary, we present a novel case of peri-adrenal PGL-induced rTCC managed with VA-ECMO and subsequent adrenalectomy. Reverse takotsubo-like cardiomyopathy is a rare and dangerous presentation of catecholamine-induced cardiomyopathy. Oestrogen deficiency may contribute to the development of cardiomyopathy via decreased activation of the ER receptor, leading to cardiomyocyte apoptosis, inflammation, and oxidative damage. The precise connection between oestrogen deficiency and rTCC is unknown. Paragangliomas should be considered when a patient has a history of episodic headaches and palpitations, oestrogen deficiency, and subsequently develops severe cardiomyopathy with a rTCC pattern and a concomitant mass on imaging. Due to the incompletely defined relationship between oestrogen deficiency and rTCC, future research should examine the histologic, pathophysiologic, and clinical implications of oestrogen deficiency in the setting of rTCC.

## Supplementary Material

ytad591_Supplementary_Data

## Data Availability

The data underlying this article are available within the text of the article. Data will be shared upon reasonable request to the corresponding author.

## Lead author biography

Steven Ajluni is a Chief Resident of the Case Western Reserve University/University Hospitals Internal Medicine Department in Cleveland, OH, USA. He has particular interests in humanistic medicine, medical education, heart failure, and interventional cardiology. He is originally from Detroit, MI, USA, and completed his medical school in Houston, TX, USA, at McGovern Medical School at the University of Texas Health Science Center at Houston. He will begin his cardiovascular medicine fellowship in July 2024 at the University of MIchigan and aims to continue his career in academic medicine as a clinician, educator, and mentor.

## Notes

**Consent:** The patient agreed to the submission of this manuscript and agrees that there is no identifying information present in this manuscript as written. The patient consents to the submission of this manuscript to the *European Heart Journal – Case Reports*.

**Funding:** None declared.
