# Supplementary material for: Paraganglioma-induced reverse takotsubo syndrome treated with extracorporeal membrane oxygenation in a young patient with a history of malignancy: a case report
Source: Eur Heart J Case Rep. 2023 Nov 24;7(12):ytad591. doi: 10.1093/ehjcr/ytad591 (PMC10733169; doi:10.1093/ehjcr/ytad591)
Supplement: ytad591_Supplementary_Data [file ytad591_supplementary_data.zip › Table 1.pdf]

Table 3. Summary of literature detailing 7 cases of severe cardiomyopathy with an rTCC pattern in the presence of an occult catecholamine secreting tumor managed by VA-ECMO

Article	Age	Gender	Presenting Symptoms	Cardiac Arrest?	Pre/Post LVEF (%)	ECG Changes	Length of ECMO/Length of Intubation (days)	Renal replacement therapy?	Length of Hospital Stay	Mortality	Management of Endocrinopathy	Urine/Serum Sympathetic Markers	Time between ECMO decannulation and surgery (days)
Zegdi et al. 2008	51	F	Shortness of breath, tachycardia and hypertension	Yes	<30/72	ST depression in inferior and lateral leads	6/8	No	NR	No	Surgical removal	Elevated/NR	30
Flam et al. 2014	46	F	Abdominal pain, shortness of breath, nausea and vomiting	Yes	15/50	Sinus tachycardia without signs of ischemia	7/14	Yes	NR	No	Surgical removal	NR/Elevated	66
Van Zwet et al. 2016	27	F	Headache, retrosternal pain, palpitations, nausea, vomiting	Yes	20/45	Non-ischemic	7/7	No	30	No	Surgical removal	NR/Normal	22
Aziz et al. 2018	59	F	Retrosternal chest pain, vomiting, hypotension	No	20/62	N/A	7/7	No	NR	No	Surgical removal	Elevated/NR	21
Kiamanesh et al. 2019	45	F	Retrosternal chest pain, dyspnea and headache	No	15/~50	Sinus tachycardia, ST segment depression II, III, V4-V6	3/NR	No	24	No	Surgical removal	Elevated/NR	14
Whitler et al. 2019	49	F	Nausea, vomiting, hypertension and tachycardia	Yes	10/60	Anterolateral and inferior ST-depressions	NR	No	NR	No	Surgical removal	NR/NR	NR
Zhou et al. 2022	69	M	Chest pain, nausea, vomiting	Yes	10/65	ST depression V4-V6	7/11	No	NR	No	Surgical removal	Elevated/NR	90
