# Supplementary figures and images for: Paraganglioma-induced reverse takotsubo syndrome treated with extracorporeal membrane oxygenation in a young patient with a history of malignancy: a case report
Source: Eur Heart J Case Rep. 2023 Nov 24;7(12):ytad591. doi: 10.1093/ehjcr/ytad591 (PMC10733169; doi:10.1093/ehjcr/ytad591)

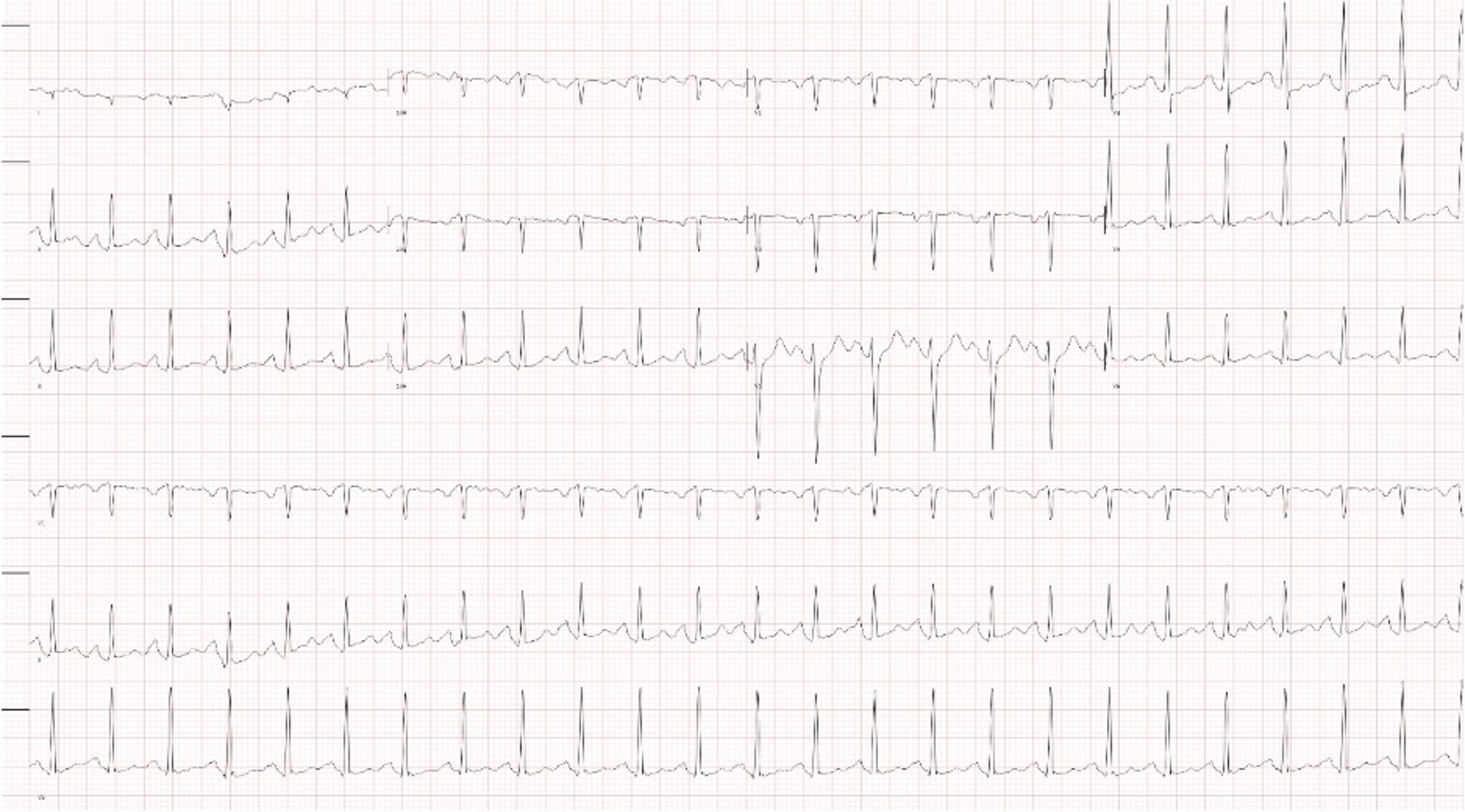

Supplement: ytad591_Supplementary_Data [file ytad591_supplementary_data.zip › Figure 1_e.tif]
